# Narrative Exposure Therapy as a treatment for child war survivors with posttraumatic stress disorder: Two case reports and a pilot study in an African refugee settlement

**DOI:** 10.1186/1471-244X-5-7

**Published:** 2005-02-03

**Authors:** Lamaro P Onyut, Frank Neuner, Elisabeth Schauer, Verena Ertl, Michael Odenwald, Maggie Schauer, Thomas Elbert

**Affiliations:** 1 vivo Uganda, Mbarara, Uganda; 2 Mbarara University of Science and Technology, Mbarara, Uganda; 3 University of Konstanz, Centre for Psychiatry Reichenau, Haus 22, Feursteinstr. 55, D-78479 Reichenau-Lindenbühl, Germany

## Abstract

**Background:**

Little data exists on the effectiveness of psychological interventions for children with posttraumatic stress disorder (PTSD) that has resulted from exposure to war or conflict-related violence, especially in non-industrialized countries. We created and evaluated the efficacy of KIDNET, a child-friendly version of Narrative Exposure Therapy (NET), as a short-term treatment for children.

**Methods:**

Six Somali children suffering from PTSD aged 12–17 years resident in a refugee settlement in Uganda were treated with four to six individual sessions of KIDNET by expert clinicians. Symptoms of PTSD and depression were assessed pre-treatment, post-treatment and at nine months follow-up using the CIDI Sections K and E.

**Results:**

Important symptom reduction was evident immediately after treatment and treatment outcomes were sustained at the 9-month follow-up. All patients completed therapy, reported functioning gains and could be helped to reconstruct their traumatic experiences into a narrative with the use of illustrative material.

**Conclusions:**

NET may be safe and effective to treat children with war related PTSD in the setting of refugee settlements in developing countries.

## Background

In the wars and armed conflicts of the past decades, children have been among the survivors who have been exposed to war or conflict-related violence. The United Nations High Commissioner for Refugees (UNHCR) recently stated that 43% of its population of concern are children under the age of 18 [1]. Mental health experts are also becoming more aware that war and conflict-related event types are among those that may result in children developing disorders of the stress spectrum, including posttraumatic stress disorder (PTSD) [2-5].

An increasingly important field of research addresses the wide-ranging negative sequelae that children and adolescents in modern post-conflict populations such as in Iraq, Kuwait, Bosnia, Rwanda, Croatia, South Africa and others may develop consequent to war and conflict violence [6-17]. Current research emphasis is now more than ever being placed on developing appropriate interventions that address the needs of survivors experiencing a range of symptoms after trauma exposure [18-29].

Given the pervasiveness of war and conflict-related trauma, especially in resource poor countries, interventions tailored to suit the circumstances of the overwhelming number of such survivors are especially in demand. However, treatment outcome studies in this field are still few. Many such interventions are derived from interventions initially developed for adults, such as cognitive behavioural therapy. Cognitive-behavioural interventions have been successfully used with school children exposed to violence, after single-incident stressors, after natural disasters as well as to treat sexually abused children [18,30-33].

Other interventions currently in use with children include psycho-pharmacological treatments, play therapy, psychological debriefing and testimony therapy [17,20,23,26,34-39]. It is notable that most approaches have not yet been tested within post-conflict populations of children and adolescents living in non-industrialized countries.

Narrative Exposure Therapy (NET) is a treatment approach that was developed for the treatment of PTSD resulting from organized violence. **vivo **developed Narrative Exposure Therapy as a standardized short-term approach based on the principles of cognitive behavioural exposure therapy by adapting the classical form of exposure therapy to meet the needs of traumatized survivors of war and torture [40-42]. In exposure therapy, the patient is requested to repeatedly talk about the worst traumatic event in detail while re-experiencing all emotions associated with the event. In the process, the majority of patients undergo habituation of the emotional response to the traumatic memory. In addition to the reconstruction of the traumatic memory, this habituation consequently leads to a remission of PTSD symptoms.

As most victims of organized violence have experienced many traumatic events, it is often impossible to identify the worst event before treatment. To overcome this difficulty in NET, the patient constructs a narration of his whole life from early childhood up to the present date while focusing on the detailed report of traumatic experiences. The focus of NET is therefore two-fold. As with exposure therapy, one goal is to reduce the symptoms of PTSD by 1) confronting the patient with memories of the traumatic event. However, recent theories of PTSD and emotional processing suggest that the habituation of the emotional processes is only one of the mechanisms that improve symptoms [43]. Other theories suggest that the distortion of the explicit autobiographic memory of traumatic events leads to a fragmented narrative of the traumatic memories. Thus, 2) the reconstruction of autobiographic memory and a consistent narrative should be used in conjunction with exposure therapy. Emphasis is put on the integration of emotional and sensory memory within the autobiographic narrative. Narrative Exposure Therapy was initially developed for adults, but has been adapted for use with children older than 8 years [43,44].

In narrative exposure procedures, children are asked to describe what happened to them in great detail, paying attention to what they experienced in terms of what they saw, heard, smelled, felt, the movements they recall and how they felt and thought at the time. Initially, the session is distressing, but as it is long enough to allow habituation, distress levels diminish towards the end and more and more details are recalled. After only four sessions of exposure, scores on intrusion and avoidance may drop significantly [43].

This study investigated the effectiveness of NET when applied to child refugees. The investigation was carried out in the context of the Nakivale mental health project, which aimed at the examination of mental health symptoms as well as the evaluation of different treatment approaches in the Nakivale refugee settlement in Uganda [45].

The first aim of this paper is to present and illustrate the procedure of KIDNET as a child-friendly treatment approach for traumatized children in post-conflict populations. In addition, we present the results of a small sample pilot test to allow the examination of the feasibility and potential efficacy of the method in a field context.

## Methods

### Ethical approval

The study protocol was approved by the Ethical Review Board of the University of Konstanz and by the Ugandan National Council for Science and Technology, Kampala.

### Participants

Six child refugees (ages 13 – 17 years; 3 girls and 3 boys) of Somali ethnic origin screened as having PTSD from a larger epidemiological survey in Uganda's Nakivale refugee settlement [45].

### Instruments and procedure

As instruments need to go through a work-intensive process of translation and validation and interpreters as well as interviewers need extensive training so that instruments are properly applied, we used the same already-validated instruments as in the adult epidemiological survey [45]. The Posttraumatic Diagnostic Scale (PDS) and the Hopkins Symptom Checklist-25 (HSCL) were administered face-to-face by trained, local, non-professional interviewers as assisted self-report interviews in order to screen for posttraumatic stress disorder and co-morbid depression [46,47]. The six children were identified as having PTSD according to the PDS. These diagnoses for both PTSD and depression were validated using the Composite International Diagnostic Interview (CIDI) version 2.1 [48] Sections K and E respectively, administered by expert clinicians with the help of extensively trained interpreters within two weeks of the initial interview.

This was done within the context of a clinical interview during which the clinicians clarified the questions for the children. The clinicians employed child-appropriate language to make sure the children understood the questions. In all six cases, the initial diagnosis was confirmed.

All six were assigned to a Narrative Exposure Therapy (KIDNET) treatment group, with treatment being offered by expert clinicians experienced in the use of NET. The post-tests were also conducted by expert clinicians and trained interpreters, again using the CIDI.

All six child patients gave verbal assent for the screening. They were then formally offered individual treatment after diagnosis, along with a brief psycho-education describing the nature and prevalence of PTSD symptoms, and what treatment would entail. A standard written rationale that has been developed for this purpose was used, the goal being to explain that PTSD-related symptoms and dysfunction are frequently consequent to multiple traumatic experiences. All six gave their assent for treatment, which only began after informed consent from the parents or guardians was granted. It was made clear that both assessment and treatment would be entirely voluntary, and no monetary or food-item inducements would be offered. In all cases, both the patients and their parents or guardians were relieved that treatment was offered.

The patients were tested again with the CIDI approximately four weeks (post-test) and nine months (follow-up) after the end of treatment.

### Treatment modality

Narrative Exposure Therapy treatment sessions lasted between 4 to 6 sessions, of between 1 – 2 hours in duration. The treatment involved one-to-one sessions with a clinician attending to an single child patient at a time. At the beginning of Session 1, the patient was requested to draw any picture that came into their minds, as the very first step. Next, the patient was given a lifeline (length of rope) and a selection of stones of varying characteristics and sizes, as well as fresh flowers of varying sizes and colours. He or she was then asked to construct his lifeline, outlining the major events using flowers for positive events and stones for negative events in a chronological order.

When he or she was quite sure about the sequence and magnitude of events, the patient was then asked to make a drawing of this lifeline, with brief titles for each event. The narrative session then began, with the patient narrating the events of his or her life starting with his or her birth, with the aid of the lifeline and the drawings. The patient used a representative object, which he or she moved at will to indicate where he or she had reached on the lifeline.

In the following narration procedure, the participant constructed a detailed chronological account of his own biography in cooperation with the therapist. The therapist's task was to document the patient's autobiography, which was corrected with each subsequent reading. Special focus of the therapy was on the transformation of the generally fragmented report of traumatic experiences into a coherent chronological narrative, and working through emotions, sensations and reactions relevant to the traumatic events. During the discussion of the traumatic life experiences, the therapist asked for current emotional, physiological, cognitive and behavioural reactions, while accompanying the patient back into the details and emotions surrounding each event, and helping the patient to reconstruct the trauma memory. The participant was encouraged to relive these reactions and emotions while reporting the events. The discussion of a traumatic event was not terminated until a habituation of the emotional reactions presented and reported by the patient occurred.

During the session and in subsequent sessions, the testimony was read back to the patients, who was asked to correct, modify or add to it until a complete document has been made of the patient's experiences. During the last session, the participant received a complete written document of his biography. The precepts of Narrative Exposure Therapy are described in detail in a manual [43].

The main innovation of KIDNET as compared to the adult version of NET is the use of illustrative material such as a lifeline (usually a length of rope or string), stones and flowers, as well as coloured drawings and role-play, to help the child reconstruct the memories of his or her experiences. Unlike with adult NET thus far, the patients were encouraged to extend the narration beyond the present, to describe their hopes and aspirations for the future, mainly with the use of flowers. This is done at the end of therapy. These hopes and aspirations were included in the drawings, lifeline and narrations as an integral part of the document. The therapist also highlighted the length of rope still left over, to illustrate opportunities and possibly improved life circumstances, despite negative past experiences. The patient was requested to construct his or her lifeline at the beginning of each session, after which narration was resumed. The therapist was also alert to detect any connection between the initial picture and the traumatic events in the storyline, especially the worst-ever event. As a final task, the therapist requested the child to draw any picture that came to their minds after handing over the document to the patient at the end of the last session, in order to compare this with the initial picture drawn.

## Results

### Two cases of Narrative Exposure Therapy

#### Case one AWH

**AWH **is a very slim young person of middle height, with active uneasy eyes that never rest. He was interviewed in March 2003 when he was 15 years old by a locally trained interviewer using the PDS. This PTSD diagnosis was confirmed within a two-week period by an expert using the CIDI, after which AWH was invited for therapy. He was very shy when he came. In fact, he approached the house then ran back into the street. After some time, he slowly approached again. Both the therapist and the interpreter put in a lot of effort to make him feel welcome and comfortable. As we later learnt, he lost both parents during the civil war in Somalia. This was without question his "worst event".

He has lived by himself since the age of 14 in the camp, but fended for himself since the age of 9. He had no family in the camp or anywhere else, as far as he knew. At the time of his parent's death in Somalia, he recalled a younger brother and sister, but he had never heard of them again. He has been a registered refugee in Uganda since 1998.

His initial drawing at the beginning of therapy shows the tiny lonely figure of a child, placed in the middle of the large white sheet of paper, nothing else. He named it " a boy".

The beginning of NET was challenging, since he seemed to recall very little of his early years as a child in Somalia. He was also very economical with words and upon being asked to place flowers for good events in his life, he found there was no event that would deserve a flower. He simply placed two stones on his lifeline, one for the day his parents were shot in front of him, one for the time he reached Nairobi as a refugee, a time when he had to struggle hard to stay alive in the absence of any aid.

After NET 1 (session 1), he did recall his early years in quite some detail and even remembered some joyful events; for one of which he later placed a flower: 'the time I used to play football with my dad in the evenings in our compound'.

After having witnessed the death of his parents and being able to escape through the back of the house, he was completely on his own. He escaped Mogadisho with a group of strangers, survived Nairobi by himself, eating left-overs at hotel garbage dumps and finally smuggled himself into Uganda by hiding in the back of a bus.

Excerpts of his story read:

I was born in Mogadisho, Somalia. I do not know my exact birthyear, I think it is 1986. I grew up with both my parents. I have a sister who is 2 years younger called Halimo and a 4 years younger brother called Mohamed. We lived in a part of town called ...

My mother had fair brown hair and skin. She was young and I loved her a lot. I was her first-born and her favourite. She even told me so. My father was of darker complexion. He was also a young man in those days. He was hard working. He had a shop close by in the market. He would usually leave in the morning and return home in the evening. Sometimes when he came home, he played with us in the evening. We played football together. Those were good times. I do not know how old I was then; I just remember that I was very young...

When I remember those days I get sad. All these memories come back and I only know what I have lost. The years went by and I used to live like this until the war broke out. I don't remember the year, but I was still young... One day we fled from home in the late afternoon...We went with a car to a place called Bal'aad, about 30 km out of town...

Eventually we went back home to Mogadisho. I must have been about 10 years by then. A few months later, the war reached us again. It was early in the morning. A group of about 10 civilian men reached came to our house. They were armed with guns...

I stood very near to my parents. I was so scared. Suddenly I heard the sound of bullets. One of the soldiers had started shooting. The moment I saw that he pulled the trigger and heard the first bullet, I panicked. I started running. I felt such great fear. I ran inside the house and tried to hide myself behind a door in one of the rooms. I was shivering, fearing, thinking, they will also come for me, they will come and kill me'. I still have a heart beat now, when I recall that moment. After some time it went quiet outside. I still stood behind the door, silent, not moving. After a while I slowly moved towards the window and peeped out. What I saw was terrible. My mother and my father had been hit by the bullets. They were both lying on the ground. My mother had fallen on top of my father. They both had blood on their clothes. My mother had blood on her face and her stomach. They were not moving anymore, they had died. Until that day, I had never seen a dead person. I felt horror. I was so afraid of them, shocked by what I saw. I only thought of running away, leaving this place. I escaped through the back of the house and jumped over the fence. This was the last time I have seen my parents and also the last time I had been in our home in Mogadisho...

While fleeing, I joined strangers in the street. So many people were trying to flee. I simply ran with them. When these people reached their destination, they branched off from the road. It was night time by then. I was alone. I hated my life. I followed the road and finally fell asleep under a bush. I had given up about life by then. I felt like I had died as well. I knew about the danger of wild animals and lions, but I did not care...

This is how I came to Uganda. When we reached Kampala I saw a group of Somalis and went to greet them. They took me in and I lived with them for a few weeks. They also showed me how to register as a refugee with UNHCR. I remember the day I came to Nakivale Refugee Camp. I was so surprised how people can live in a place like this. I stayed with the Somali family that found me in Kampala for about two years in the camp. Finally, Red Cross helped me to build my own house in 2000, I was about 14 years then. Since then I live alone. I started going to school when I came to Nakivale. I will graduate from P7 at the end of this year. I have learnt how to live by myself; I can do everything by myself. I never ask for help. No one can help me anyway. I have never heard about my brother and sister again. Whether they are still alive and if so, where I could find them. But now I am ready to look for them.

As mentioned before, he has been surviving on his own since the age of 9 years. This has probably led to his enormous shyness but also an amazing sense of self-reliability. 'I can do everything by myself. I never ask for help. Anyway who could help me?' There is also a strong feeling of sadness and loneliness around him that is very moving. Our local translator Haji, who is also trained as a NET therapist, was moved to free flowing tears at several moments of AWH's life recall, specially when he spoke about his loneliness and desperation.

AWH is a smart boy, so he frequently challenged the concept of therapy in the beginning. 'It won't make my parents come back, and it won't change my life situation'. AWH has never really entered a state of expressing strong emotions like crying or anger during therapy. He said he could not cry, also not by himself. There were however visible signs of emotional processing like tears and strong heartbeat especially during NET 1 and 2.

AWH reported having strong PTSD symptoms when he started therapy, especially continuous nightmares and flashbacks, mainly related to the day his parents were killed. Re-experiencing actually increased, he said, once therapy was started. After NET 4 he talked of having fewer symptoms. In his last session AWH talked about wanting to try and trace his family with the help of the Red Cross. He was especially interested in finding his two younger siblings.

When the sum of symptoms were counted according the CIDI-K section, AWH had a sum score of 12 in the CIDI pre-test. This had decreased to 8 in the CIDI post-test immediately after the end of therapy, and 9 at the nine-month follow-up. At the nine-month follow-up, AWH was looking well-dressed, and had put on both height and weight. He had completed his primary school education at the camp primary school and spoke confidently of his plans. He had also joined the camp soccer team and played along with the group now every evening, a significant behavioural change, given that he previously would not even talk or socialize with others. He was not more friendly than usual, but admitted significant symptom reduction to an expert evaluator who had never seen him before.

#### Case two – UG

UG was a pretty seventeen-year old girl in March 2003 when she was interviewed by a locally trained interviewer. She looked, however, visibly ill and strained -quite unlike a normal happy young woman. She complained of constant headache and pain in her eyes. UG's PTSD diagnosis was confirmed within a two-week period by an expert using the CIDI.

UG did not wait to be offered treatment, but came herself to the project centre and requested for help. In her own words, "I have been to all the doctors but they have done nothing for me. All they give me are painkillers. I had to drop out of school because of my headaches and pain in my eyes." When the treatment protocol was explained to her, she was enthusiastic about relating her experiences and readily gave her assent. Her mother was quick to give her consent. She said she would try anything that would help her daughter. In her own words, ' My daughter has not been her real self for a long time.'

At the beginning of NET 1, UG drew a picture of the Somali flag. Asked to explain, she said she loved her homeland and hoped to go back there one day when there was peace. While laying her lifeline, UG included a happy family life in Somalia till the age of 4, her arrival in Uganda after a long and difficult flight itinerary and being accepted as a refugee by the UNHCR as the flowers in her life. The stones in her life represented the worst event, when her older brother was killed, her sister was severely injured and her two younger brothers were lost, never to be seen again, to date. Other stones symbolised flight difficulties such as extreme hunger, and a failure to access a solution for her headaches and eye pain, which she has had since the worst event.

In subsequent NET sessions, UG talked in detail about these events. Excerpts from her story read:

I was born on August 26th 1986 in Mogadishu.... I had six brothers, two younger than me and two sisters older than me. We were a very happy family. As a big family, we conversed a lot and made jokes. We were very happy with our father. He made us laugh and brought us presents whenever he went anywhere. This was a happy time till I was 4 years old...

One day,... we heard guns and bullets firing and we were excited. My father ran from work and picked up the other children from school and came back home with them. Some people came to chase us away from our house because we were a minority clan (Madiban). They were from the Hawie (Habrigidir). They were men, very many. They told us to leave everything and flee from the house. They did not beat us. I was still a child, with a soft head. I heard the bullets and started vomiting and fell down. My mother picked me up and put me on her back. We all left the house...

On the way, we met very many militia men dressed as army men. They told us to lie down on the ground. I was still crying. The rest were silent. One of them knocked me with the butt of the gun on the soft part of my head. Then I kept quiet. They wanted to kill everyone...

They put us in a house for security purposes. A heavy gun was shot near the house. There was another house near our house. They shot this house and the fragments reached our house. Some people were killed in our group, including my brother. He was older than me. He was called Mahad. He was in his school uniform. He fell down on his stomach. I did not see him fall but I saw the blood. My sister Khadija was lying down when the fragments hit her. I did not see any blood, but the fragments went into her stomach and she was hurt in her stomach. My father thought she was dead, but people said she was still alive and she was still talking. He went to her and told her to get up. She could not get up. My father carried her to the hospital. Two of my brothers, Abdirahman aged four years, and Abdirasaq aged three years, disappeared in that group as everyone ran away. They were my two younger brothers. My dead brother Mahad was left there. We never saw my two brothers who disappeared again. So many other people died as well...

After one year, we came back to Mogadishu. We found our father and sister in the hospital. They first stayed in the hospital for some time, then moved to a house. My sister had been operated on. My father had become a bit deaf because of the gunshot.

We decided to leave Somalia and go somewhere else more secure. We left Somalia with nothing. My mother, my father, my injured sister Khadija, and the other six children...

We went to Kampala. My mother was advised to go to the UNHCR and got a mandate. My sister Shamso and my cousin sister Fardosa also got separate mandates.... In 1997, we were resettled in Nakivale camp...

I hit my head down and I felt the pain of the gun again. I was not hurt but I got a terrible headache for three days. I went to the doctor but I did not get any assistance except Panadols and eyedrops. Since then, I was unable to go to school because the headaches increased. My eyes had begun paining from birth, but they increased with the headaches.

I am still here but I hope for a better future and a happier life e.g. to be resettled elsewhere, to have an education, to have a happy family of my own and to make contact with my lost loved ones.

Among her hopes and aspirations for the future, UG hoped to trace her missing brothers under the auspices of the Red Cross, as well as re-enter the education system. She also hoped to one day have a happy family of her own

In the final picture at the end of therapy, she drew a happy family inside a nice house, living in peace.

UG had a CIDI pre-test sum-score of 16; immediately after treatment, this reduced to 12 in the CIDI post-test and 1 at the nine-months follow-up. The nine months follow-up found UG no longer in the camp but in Kampala, the capital city of Uganda. She looked happy and cared about her appearance. She said she had no more headaches, and little eye problems. In her own words, "I feel like a newborn child," she told the blind evaluator. She had moved away from the camp to explore possibilities for further education, resettlement and to seriously try to trace her brothers. She also reported joining other adolescents when they were out to socialize. "My biggest dream is to play soccer myself, but as a girl, I would never be allowed. But at least I go and cheer to the boys when they play." She says her family members, especially her mother, have all noticed the difference in her and keep commenting on the improvements she has made. She is more like any healthy young woman.

### Pilot study

**Figure 1:**
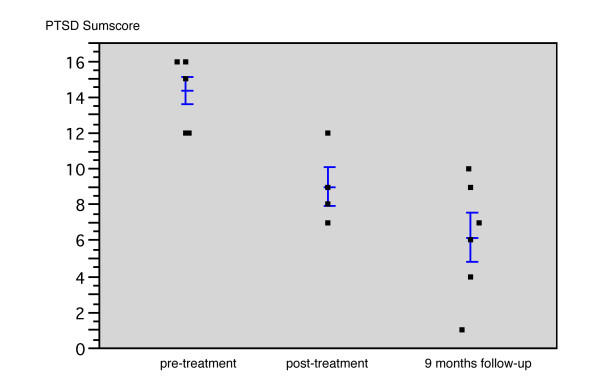
**Scatterplot of the sum of PTSD symptoms as recorded by the CIDI-K section across therapy**. Both a mixed model ANOVA and a Friedman test confirmed a significant reduction of symptoms between time intervals.

All the patients accepted and completed treatment. Two patients were unavailable for the post-test as they had left the settlement after treatment. At the 9-months follow-up, all children could be tested again, as the investigation included a testing in other regions to which Somali refugees had moved. At baseline, all six had moderate to severe PTSD according to the CIDI (*M *= 14.3, *SD *= 1.9). Scores dropped to *M *= 9.0 (*SD *= 2.2) at post-test and again to *M *= 6.2 (*SD *= 3.3) at 9-months follow-up (see Figure 1 and Table 1). A mixed model (random factor: Subject, fixed factor: Time, missing data: restricted maximum likelihood), that allows the inclusion of all six cases in the analysis, indicated a significant reduction of symptoms across time; F (2,5) = 15.45, *p *< 0.01. This result was confirmed by a Friedman-test using row-wise-exclusion of the two missing cases: χ^2 ^(df = 2) = 6.50, *p *= 0.039. The analysis at individual level showed that the symptom scores of each individual patient decreased in the period between the pre-test and the follow-up period.

**Table 1:** The participants' individual scores on the PTSD section of the Composite International Diagnostic Interview at the timepoints pre-treatment, post-treatment and 9-months follow-up.

Code	Age	Sex	CIDI pre	CIDI post	CIDI follow-up
AMA	16	female	12	9	7
ABJ	15	male	16		6
HI	13	female	15	7	4
DMM	16	male	15		10
AWH	15	male	12	8	9
UG	17	female	16	12	1

M (SD)	15.3 (1.4)		14.3 (1.9)	9.0 (2.2)	6.2 (3.3)

After nine months, four of the six patients no longer met the criteria for PTSD. The remaining two both had borderline scores.

Before treatment, four of the six patients presented with clinically significant depression as they fulfilled DSM-IV criteria for major depression according to CIDI interview. None of the subjects met the criteria for clinically significant depression according to the CIDI at the post-test or the 9-months follow-up.

## Discussion

In this pilot trial we tried to get a first impression on the efficacy and safety of KIDNET, a short-term treatment approach for the therapy of traumatized adolescents.

Results showed an important reduction in posttraumatic symptoms, as early as the post test. At the 9-months follow-up, 2 of the 6 patients still fulfilled PTSD criteria, but now at borderline levels and with less functional impairment. Clinically significant depression has remitted to non-clinical levels in all four adolescents who had presented with depression in the pre-test.

This study presents with the limitations of pilot studies with small sample size. Symptom changes cannot be causally attributed to treatment, but might also be caused by spontaneous remission. Nevertheless, given the cross-sectional data available, in particular, the high prevalence of PTSD in the Somali refugee population (with nearly 50% [45]), it seems unlikely that spontaneous remission has occurred at this rate. Otherwise, the high prevalence would be difficult to explain, as the PTSD should have remitted earlier.

Neither the clinical impression nor the symptom scores indicated a worsening of the symptoms in any of the patients. Therefore, this pilot study suggests that NET might be used effectively as a short-term treatment with child patients even in the unsafe conditions of a refugee camp in an African country. A possible adjustment would be to allocate more sessions where needed, such as where traumatic events were particularly severe or numerous.

The case reports illustrated that the child patients were able and willing to narrate their traumatic experiences, especially with the aid of illustrative material. There is also evidence that children can recall details of traumatic events that occurred when they were younger. Teenaged children are also able to comply with short-term treatment approaches such as the version of NET presented here.

It is noteworthy that this group of children had multiple and very severe war events and yet showed a clear benefit from treatment with NET. This encourages research into the effectiveness of KIDNET with other child trauma populations such as child PTSD after single stressors, after natural disasters or after accidents. More pressing of course, are randomized controlled trials comparing KIDNET to existing therapies for children. Any such comparisons between different groups of children has to await further research.

It is often a matter of survival that mental function is restored to an extent that the survivor can cope with the daily stressors and that children actively take advantage of scholastic opportunities and ultimately strive for finding ways out of the camp.

In this study, the therapies were carried out by highly trained experts. Given the large numbers of possibly traumatised youth in post-conflict low-income areas worldwide, treatment costs would be prohibitive unless further research can increase the ease with which non-professional paramedics can acquire adequate therapy skills through rigorous short-term training and supervision.

## Conclusions

KIDNET proved to be a feasible method for the treatment of traumatized children in an African refugee settlement. The clinical observations and the assessed reduction in symptoms should approve KIDNET to be tested in further controlled trials.

## Competing interests

The author(s) declare that they have no competing interests.

## Authors' contributions

The study was designed by LPO, FN, MO, MS and TE. PLO, VE, and ES carried out the treatments and the assessments, FN and MS supervised the treatments. Data was analyzed by FN and LPO. LPO drafted the manuscript, all authors revised the manuscript and approved the final version.

## Pre-publication history

The pre-publication history for this paper can be accessed here:


